# Gene Expression and MicroRNA Expression Analysis in Small Arteries of Spontaneously Hypertensive Rats. Evidence for ER Stress

**DOI:** 10.1371/journal.pone.0137027

**Published:** 2015-09-10

**Authors:** Teresa Palao, Karl Swärd, Aldo Jongejan, Perry D. Moerland, Judith de Vos, Angela van Weert, Silvia M. Arribas, Gergely Groma, Ed vanBavel, Erik N. T. P. Bakker

**Affiliations:** 1 Department of Biomedical Engineering and Physics, Academic Medical Center, Amsterdam, The Netherlands; 2 Department of Experimental Medical Science, Lund University, Lund, Sweden; 3 Bioinformatics Laboratory, Academic Medical Center, Amsterdam, The Netherlands; 4 Departamento de Fisiología, Facultad de Medicina, Universidad Autónoma de Madrid, Madrid, Spain; The University of Manchester, UNITED KINGDOM

## Abstract

Small arteries are known to develop functional and structural alterations in hypertension. However, the mechanisms of this remodeling are not fully understood. We hypothesized that altered gene expression is associated with the development of hypertension in mesenteric arteries of spontaneously hypertensive rats (SHR). Three sublines of SHR and normotensive Wistar Kyoto rats (WKY) were studied at 6 weeks and 5 months of age. MiRNA and mRNA microarray experiments were performed and analyzed with bioinformatical tools, including Ingenuity Pathway Analysis (IPA). Principal component analysis showed a clear separation in both miRNA and mRNA expression levels between both ages studied, demonstrating strong age-related changes in expression. At the miRNA level, IPA identified differences between SHR and WKY related to metabolic diseases, cellular growth, and proliferation. The mRNAs differentially expressed between SHR and WKY were related to metabolism, cellular movement and proliferation. The most strongly upregulated gene (9.2-fold) was thrombospondin 4 (*Thbs4*), a protein involved in the endoplasmic reticulum (ER) stress response that activates transcription factor 6α (ATF6α). ATF6α downstream targets were also differentially expressed in SHR vs. WKY. Differential expression of THBS4, the cleaved form of ATF6α, and two of its targets were further confirmed at the protein level by western blot. In summary, these data revealed a number of genes (n = 202) and miRNAs (n = 3) in mesenteric arteries of SHR that had not been related to hypertension previously. The most prominent of these, *Thbs4*, is related to vascular ER stress that is associated with hypertension.

## Introduction

Despite ample treatment options, hypertension remains one of the most important risk factors for cardiovascular disease. Blood pressure levels are tightly related to peripheral resistance, which is mainly determined by small arteries and arterioles in the vascular bed. These vessels, referred to as resistance arteries, show structural (remodeling) and functional changes in hypertension, that may increase peripheral resistance and thereby blood pressure. Such changes include inward remodeling, hypertrophy, and impaired endothelium-dependent dilation [1].

At the level of the resistance arteries, few studies addressed alterations in gene expression in a genome wide manner. Thus, previous studies compared mRNA expression between spontaneously hypertensive rats (SHR) and its normotensive control, the Wistar Kyoto rat (WKY), in kidney [2,3], adrenal gland [4], vascular smooth muscle cells [5], and in saphenous arteries[6]. One study compared collateral resistance arteries from the mesenteric vascular bed of SHR and WKY [7]. None of these studies included the more recently identified miRNAs, which add another layer of regulation. Therefore, in the current study we tested the hypothesis that gene expression is altered in the development of hypertension in mesenteric arteries of SHR as compared to WKY. To address this hypothesis, we used miRNA and mRNA microarrays, as well as bioinformatical analysis.

As a previous study by our group identified differences in remodeling between sublines of SHR and WKY [8], three sublines of SHR and WKY were used to identify genes that are consistently up or down regulated with hypertension. In addition, to study the change in the expression pattern during hypertension development, two time points where chosen: 6 weeks of age, which is considered as a pre-hypertensive stage, and 5 months of age, where hypertension is established [9]. The data showed differences in miRNA and mRNA expression between SHR and WKY, as well as between both ages in SHR and in WKY. A number of genes, pathways and miRNAs that were found to be differentially expressed in mesenteric arteries of SHR vs. WKY have not been related to hypertension previously.

## Materials and Methods

### Animals

Male SHR and WKY rats at 6 weeks and 5 months of age (n≥5 per sub-line and time point) were obtained from three different locations. The SHR/NCrl, WKY/NCrl, and stroke-prone SHR animals (SHRSP/A3NCrl) rats were obtained from Charles River, the Netherlands. The SHR/NHsd and WKY/NHsd rats were derived from Harlan UK, (Bicester, U.K.) and bred at the local animal facility of the Universidad Autónoma de Madrid, Spain. WKY/NTac rats were obtained from Taconic Farms (Germantown, NY, USA).We studied male rats in order to minimize variations in gene expression related to the estrous cycle in female rats. All experiments were approved by the Committee for Animal Experiments of the Academic Medical Center, Amsterdam. Data on blood pressure and vascular remodeling in this set of animals has been published previously [8].

### Tissue collection

Animals were anesthetized with isoflurane (5%), weighed, heparinized, and blood was taken by a heart puncture. Animals were then killed by decapitation. The intestines were removed in block and placed in chilled MOPS buffer. Second- and third-order branches of the mesenteric artery were dissected using a stereomicroscope. From each animal, two vessels were cleaned from adipose tissue and blood, leaving the adventitia and endothelium intact. They were stored in sterile tubes at -80°C for miRNA and mRNA analysis. Additional vessels were taken for RT-PCR confirmation.

### Microarray analysis

Gene expression was analyzed in WKY and SHR sublines at two ages (6 weeks and 5 months). For each subline, vessels from five animals were pooled, yielding 12 samples in total. Total RNA including microRNAs was isolated using the miRNeasy Micro Kit (Qiagen) according to the manufacturer’s instructions. Integrity of the isolated RNA was evaluated using the RNA 6000 Pico kit and Small RNA kit on a BioAnalyzer system (Agilent Technologies).

For whole transcriptome analysis, 100 ng total RNA per test sample was combined with Spike A and amplified incorporating Cy3 according to the “Agilent Two-Color Microarray-Based Gene Expression Analysis Guide” version 6.6 using the Quick Amp Labeling Kit (Agilent Technologies). As common reference an equimolar pool of all test samples was made of which 100 ng was amplified as described above with the exception that Spike B was included and Cy5 was used for labeling. Synthesized cyanine-dye labeled antisense RNA (aRNA) was purified using the RNeasy MinElute Cleanup Kit (Qiagen) before measuring yield and dye incorporation on a NanoDrop ND-1000. Labeled test samples and common reference were combined and hybridized on SurePrint G3 Rat GE 8x60K microarrays (G4858A-028279, Agilent Technologies) according to the manufacturer’s instructions.

For miRNA expression analysis, 100 ng of total RNA including microRNAs was labeled with Cy3 as described in the “miRNA Microarray System with miRNA Complete Labeling and Hyb Kit Manual” version 2.2 (Agilent Technologies) with the inclusion of spike-ins and the optional desalting step with spin columns (Micro Bio-Spin 6, Bio-Rad). Labeled samples were hybridized on rat 8x15k miRNA microarrays (G4471A-046066, Agilent Technologies), which are based on Sanger miRbase release 19.0. After washing, the arrays were scanned in an ozone-free room with an Agilent DNA microarray scanner (G2565CA, Agilent Technologies). Data was extracted with Feature Extraction software v10.7.3.1 (Agilent Technologies) using the miRNA_107_Sep09 protocol for miRNA microarrays.

Data analyses were carried out with Bioconductor packages in the statistical software package R (Version 3.0.0). Separate analyses were performed for the mRNA and miRNA arrays. All arrays were subjected to a set of quality control checks, such as visual inspection of the scans, checking for spatial effects through pseudo-color plots, and inspection of pre- and post-normalized data with box plots, density plots, ratio-intensity plots, heatmaps and principal component analysis using the arrayQualityMetrics package. Pre-processing and summarization of miRNA probe-level data was done using a modified version of the robust multi-array average (RMA) method with background correction, implemented in the AgiMicroRna package. This pre-processing method has been shown to have better precision than the one recommended by Agilent [10]. Only non-control miRNAs detected on at least one microarray according to Agilent Feature Extraction software were included in the further analysis. Background correction of the mRNA arrays was performed using the “normexp” method [11] with an offset of 15 to adjust the foreground signal without introducing negative values (limma package). The resulting log ratios were transformed using intensity-dependent loess normalization (limma package). Control probes were down-weighted to zero in the normalization step and not included in the further analysis. Normalized log-ratios for replicate probes were averaged. Probe annotation was downloaded from https://earray.chem.agilent.com/earray/catalogGeneLists.do?action=displaylist (028279_D_GeneList_20130204.txt).

For both experiments a 2x2 factorial design was used with strain (WKY/SHR) and age (6 weeks/5 months) as factors. Differential expression was assessed by fitting gene-wise linear models with a factor containing the 4 conditions as explanatory variable. In the mRNA experiment four samples were hybridized twice. These technical replicates were handled by estimating a common value for the intra-replicate correlation and including it in the linear model. To find genes with differential expression between two conditions, we employed a moderated t-test and adjusted the resulting p-values to correct for multiple hypothesis testing using the Benjamini-Hochberg false discovery rate (FDR). For unsupervised analysis of the mRNA data, normalized log-ratios of technical replicate arrays were averaged. Dendrograms were generated with Pearson correlation as distance measure, complete linkage as agglomeration method (function hclust using the 100 miRNAs with highest variance and the genes with an inter-quartile expression range larger than 0.6, respectively.). Principal component analysis was performed on scaled data (function prcomp).

The miRNA and mRNA data have been deposited in the NCBI Gene Expression Omnibus in a MIAME compliant format and are accessible under GEO Series accession number GSE53364.

Further analysis of the microarray data was performed to analyze the expression of Atf6α target genes. The list of targets was obtained from Adachi et al [12] and a ROAST gene set test [13] was performed to determine whether Atf6 targets were either up or down regulated using 10,000 rotations.

### Ingenuity Pathway Analysis (IPA)

IPA software (Ingenuity Systems, http:/www.ingenuity.com), based on gene ontology (GO) and published literature, was used to identify biological processes, canonical pathways, molecular functions and genetic networks where the differentially expressed miRNAs or mRNAs from our microarray data could interact. For the studies, the core analysis tool was used, and the cutoff log ratio was set to 0.5, so that genes/miRNAs with a log2(fold change)>0.5 or <-0.5 were selected. As cell lines, smooth muscle cells, endothelial cells and fibroblasts were chosen in the settings. Top canonical pathways, molecular functions and networks are presented as generated by the software.

### RT-PCR

Quantitative real time PCR was used to evaluate the expression levels of 6 miRNAs and 10 mRNA that emerged as the most up- or down- regulated in the microarray experiments. Samples of mesenteric arteries were obtained from the same animals from WKY/NTac and SHRSP/A3NCrl sublines used for the microarray study. RNA extraction was done using the miRCURY™ RNA Isolation Kit (Exiqon) according to the manufacturer’s instructions. RNA integrity and quantification was evaluated using a RNA 6000 Pico Kit in a Bionalyzer system (Agilent Technologies). RNA with RNA integrity numbers (RIN values) > 7.0 was used for further experiments.

For the miRNA, the RNA (5ng/μL) was transcribed to cDNA using the Universal cDNA synthesis kit II–miRCURY microRNA PCR (Exiqon) following the manufacturer’s instructions. Then, the cDNA was used for the RT-PCR using the Exilent SYBR Green master mix (Exiqon) and miRCURY LNA Universal RT microRNA PCR primers set (Exiqon) for the following miRNAs: rno-miR-29b-3p, rno-miR-31a-5p, rno-miR-542-5p, rno-miR-31a-3p, rno-miR-146a-5p, rno-miR-132-3p and U6 as reference miRNA. The PCR program started with an activation/denaturalization step at 95°C followed by 40 amplification cycles of 10 seconds at 95°C and 60°C for 60 seconds. For mRNA, the RNA (500 ng) was reverse transcribed into double stranded cDNA using the Ovation Pico WTA System V2 kit (NuGEN) following the manufacturer’s instructions. The template cDNA was amplified using specific forward and reverse primers for every gene (Table 1), in combination with SensiFAST SYBR & fluorescein kit (Bioline). The PCR program consisted of an initial heating step (95°C) of 3 minutes to activate the polymerase. Subsequently, 40 amplification cycles of 5 seconds at 95°C and 30 minutes at 65°C allowed denaturation of the DNA and DNA synthesis, respectively, and were followed by a final step of 1 min at 95°C and 1 minute at 65°C. In both cases, after the amplification cycles, a dissociation curve was performed by measuring fluorescence from 65°C until 95°C in steps of 0.5°C. Fluorescence was measured with a iCycler iQ™ detection system (Bio-Rad) and the data was analyzed with myiQ 2.0 software (Bio-Rad). Gene expression was normalized to the reference gene P0 (ribosomal protein large P0). Every experiment included duplicate control reactions and all genes were determined for every sample.

**Table 1:** Real-time PCR primers used for validation of microarray data.

Gene symbol	Gene name	Primer forward/reverse
***P0***	Ribosomal protein large P0	CACAGAAGGGTCCTGGCTTTGTCTGTG
		CGCAAATGCAGATGGATCG
***Pcmtd1***	Protein-L-isoaspartate(D-aspartate) O-methyltransferase	AGCCTTCAGAGCCATTGACC
	domain containing 1	CGCATAATCTGTGTCAACTGATCC
***Ano1***	Anoctamin 1, calcium activated chloride channel	GAGATCGGCATCCCGAAGAT
		GTGCATGGTCCCGTTTTGAC
***Enpp1***	Ectonucleotide pyrophosphatase/phosphodiesterase 1	GTGGTACAAAGGACAGCCGA
		GCCTTGATGACCTCACTGCT
***α-globin***	Alpha Globin	CTGCTGAAATTGGCGCAGAG
		GCAGTGGCTCAGGAACTTGAA
***Thbs4***	Thrombospondin 4	CAGAGCCACACAGAGGCTGTC
		GCAGAAGGTCAAAGACCTGGG
***LOC302022***	Similar to nidogen 2 protein	AGGTCCGAGTGGGCGTCT
		GAGATGATCCCATTGGTGCCC
***Dio3***	Deiodinase, iodothyronine, type III	CACGGGGAACCGTCAAAAAC
		AGTCCTAGCCTCCCTCTTGG
***Esm1***	Endothelial cell-specific molecule 1	CCAAACAGAGCGCGATGC
		CCAGGATCAGCGCGGATTTA
***Mettl24***	Methyltransferase like 24	CGCCTCTACTCTTTAGGGCTG
		GGCACTTTCCAGATCTGCCT
***Sltrk5***	SLIT and NTRK-like family, member 5	AGTACAGCGTCTATGGGGGT
		CCTCCACGGAATTGCCTTCT

Gene symbols, names and sequences of the primers used for the validation of the microarray data.

### Immunohistochemistry

Segments of mesenteric arteries were embedded, frozen and cut into 5μm sections. The sections were then fixed in methanol 100% for 20 minutes. Next, a blocking step consisting of a 45 minutes incubation with 3% bovine serum albumin (BSA) + 0.005% triton X was applied at room temperature. After that, sections were incubated overnight at 4°C with the primary antibody, rabbit anti-rat thrombospondin 4 (Santa Cruz sc-7657-R; 1:100). Samples without primary antibody were used as negative control. After washing, the samples were incubated for 45 minutes at room temperature with secondary antibody, a goat anti-rabbit Cy3-labelled (Brunschwig 111-165-144) in a 1:300 dilution in 1% BSA. Incubation with bisbenzimide (3.5 mg/ml) at 1:100 dilution during 3 minutes was used to visualize the nuclei. Sections were covered with DAKO fluorescent mounting medium (DAKO–S3023) and coverslipped.

### Western Blot

Frozen aortae were pulverized in a Tissuelyser LT (Qiagen). Tissue powder was dissolved in sample buffer (62.5 mM Tris-HCl pH 6.8, 2% SDS (W/V), 10% (V/V) glycerol, 5% (V/V) mercaptoethanol) containing phosphatase and protease inhibitors (Sigma, St. Louis, USA). Protein concentration was determined using the Bio Rad DCprotein assay and 20 μg protein was loaded in the wells of precast AnyKD gels (TGX Criterion, Bio Rad, Hercules, CA, USA). Following separation in Tris-Glycine buffer, proteins were transferred to membranes (0.2 μm nitrocellulose) using the Turboblot system (Bio Rad), blocked in casein buffer (Bio Rad), washed (20 mM Tris-HCl, 0.5 M NaCl, 0.05% (v/v) Tween 20, pH 7.5), and incubated with primary antibodies raised against THBS4 (sc-390734, Santa Cruz, 1:100;), ATF6α (ab11909 abcam, 1:250), MANF (ab67271 abcam, 1:500) and PDI (#3501 Cell Signaling, 1:1000), all added directly to the blocking buffer. After washing and incubation with horseradish peroxidase-conjugated secondary antibodies (Cell Signaling Technology, Danvers, MA, USA) blots were developed using West Femto substrate (Pierce, Rockford, IL) and captured using the Odessey Fc instrument (LI-COR Biosciences). Because the loading controls tested changed in hypertension vs. normotension, bands were normalized to protein remaining on the gels after transfer, as shown previously [14]. Protein was detected using Biosafe Coomassie G-250 stain.

### Statistical analysis

Statistical analysis for the microarray was explain in the microarray analysis methods section. For other parameters, the software GraphPad Prism 5 (GraphPad Software Inc.) was used. In the case of the PCR experiments performed for validation of the microarray data, differences between WKY and SHR were tested using Mann–Whitney U test.

## Results

### Principal component analysis

**Fig 1:**
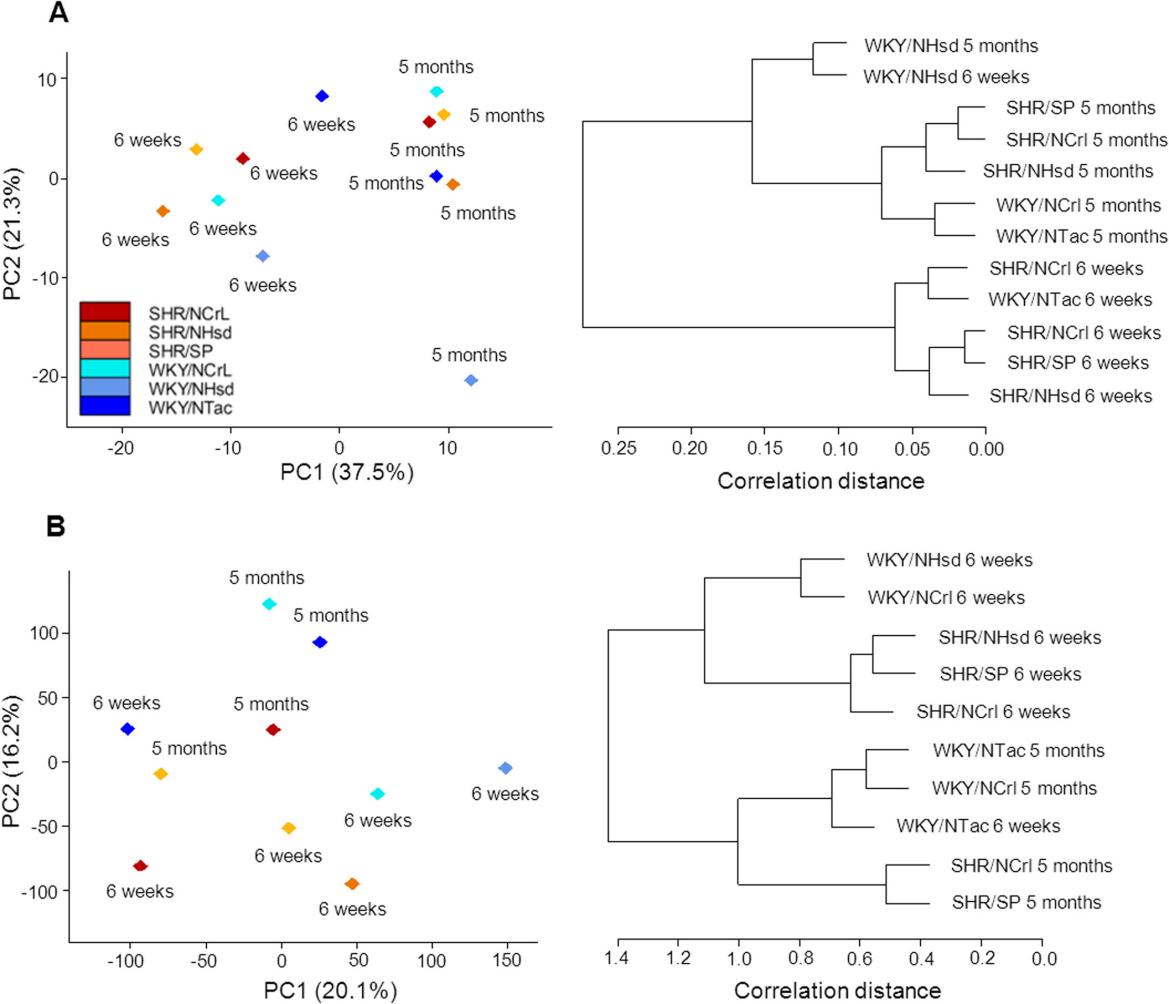
Principal component analysis and hierarchical clustering. miRNA (panel A) and mRNA expression (panel B) were analyzed in mesenteric arteries from WKY and SHR sublines at 6 weeks and 5 months of age. A clear clustering of age groups was observed, indicating strong age-related gene expression. Note that in panel A, the WKY/NHsd (5 months) stands out. In panel B, WKY/Tac at 6 weeks of age clusters with the WKY groups of 5 months of age. WKY and SHR less clearly separate, indicating a weaker relationship of gene expression with blood pressure. PC: principal component. Numbers in brackets: contribution of the principal component to variability in the data, given as percentage.

We analyzed miRNA and mRNA expression in mesenteric arteries of WKY rats and SHR at 6 weeks and 5 months of age. These time points were chosen with the objective of representing a pre-hypertensive stage and a phase of established hypertension. During the analysis, all miRNA samples passed quality control. However, in the mRNA experiment, two out of the twelve samples did not pass quality control, and were therefore excluded from further analysis (WKY/NHsd and SHR/NHsd at 5 months of age). Principal component analysis and hierarchical clustering of both miRNA and mRNA expression showed a clear separation between young and mature rats, indicating an overall pattern of age-related changes in expression (Fig 1). WKY and SHR were less clearly separated in the principal component analysis and hierarchical clustering, indicating a weaker relationship of miRNA and mRNA expression with hypertension than with maturation.

### Differential expression

**Fig 2:**
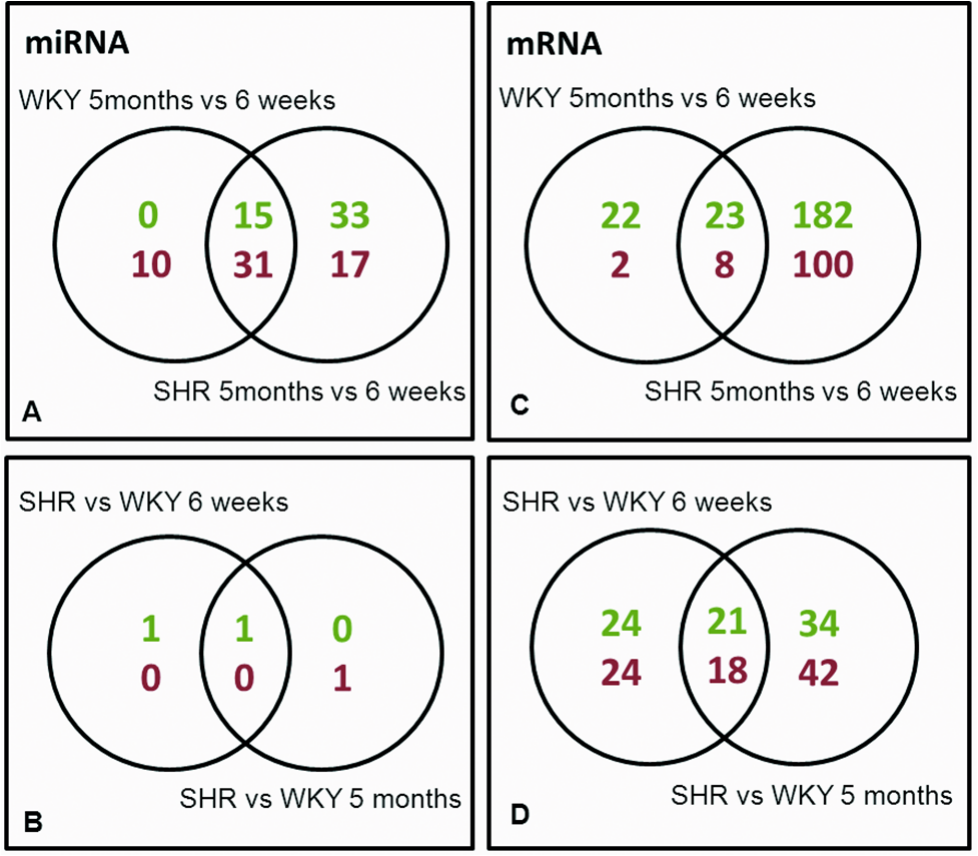
Differences in miRNA and mRNA expression. Venn diagrams showing the number of miRNAs (A, B) and mRNAs (C, D) that were significantly different in each comparison. Upper numbers (green) indicate the number of up-regulated genes; lower numbers (red) indicate number of down-regulated genes.

From a total number of 307 miRNAs that were tested, WKY showed significant differential expression (FDR<0.05) of 56 miRNAs when comparing young (6 weeks) and mature (5 months) rats (Fig 2A). In SHR, 96 miRNAs were significantly differentially expressed upon maturation. Of these, 46 miRNAs were shared between WKY and SHR (Fig 2A). The most strongly up- and down-regulated miRNAs are listed in Table 2. A direct comparison of WKY and SHR miRNA expression profiles showed only few differences between WKY and SHR. Only miR-31a-3p and miR-31a-5p were significantly upregulated in SHR at both 6 weeks and 5 months of age. In addition, at 5 months of age a significant decrease in the expression of miR-146a-5p was found in SHR as compared to WKY (Fig 2B).

**Table 2:** List of most strongly up- and down-regulated microRNAs.

**5 months vs. 6 weeks**					
**WKY**			**SHR**		
	**fold**			**fold**	
**miRNA**	**array**	**PCR**	**miRNA**	**array**	**PCR**
rno-miR-29b-3p	**4.1**	2.2*	rno-miR-29b-3p	**4.2**	**4.2**
rno-miR-29a-3p	**2.5**		rno-miR-132-3p	**2.9**	**2.3**
rno-miR-29c-3p	**2.3**		rno-miR-34a-5p	**2.6**	
rno-miR-29c-5p	**2.0**		rno-miR-29a-3p	**2.6**	
rno-miR-194-5p	**1.9**		rno-miR-29c-5p	**2.4**	
rno-miR-222-3p	**1.8**		rno-miR-29c-3p	**2.4**	
rno-miR-598-3p	**1.8**		rno-miR-145-3p	**2.1**	
rno-miR-7a-5p	**1.8**		rno-miR-222-3p	**2.1**	
rno-miR-101a-3p	**1.7**		rno-miR-598-3p	**2.0**	
rno-miR-192-5p	**1.7**		rno-miR-30c-2-3p	**2.0**	
rno-miR-322-3p	**0.5**		rno-miR-434-3p	**0.3**	
rno-miR-127-3p	**0.4**		rno-miR-450a-5p	**0.3**	
rno-miR-450a-5p	**0.4**		rno-miR-299a-5p	**0.3**	
rno-miR-181c-5p	**0.4**		rno-miR-411-3p	**0.3**	
rno-miR-411-3p	**0.4**		rno-miR-127-3p	**0.3**	
rno-miR-299a-5p	**0.4**		rno-miR-136-5p	**0.3**	
rno-miR-322-5p	**0.3**		rno-miR-379-5p	**0.2**	
rno-miR-379-5p	**0.3**		rno-miR-542-3p	**0.2**	
rno-miR-542-3p	**0.3**		rno-miR-322-5p	**0.2**	
rno-miR-542-5p	**0.2**	0.06*	rno-miR-542-5p	**0.1**	0.02*
				****	
**SHR vs. WKY**					
**6 weeks**			**5 months**		
rno-miR-31a-5p	**5.8**	**5.5**	rno-miR-31a-3p	**2.8**	7.8*
rno-miR-31a-3p	**3.5**	16.9*	rno-miR-146a-5p	**0.5**	0.3*

The upper part lists miRNAs that are differentially expressed upon maturation in WKY and SHR (5 months vs. 6 weeks; adjusted P-value <0.05). Maturation shows many similarities in WKY and SHR, with some exceptions such as miR-132-3p. The lower part of the table shows a direct comparison between SHR and WKY, which yielded only 3 miRNAs that were significantly differentially expressed. Fold change obtained during PCR confirmation is also shown (* p <0.05, n = 5–6).

At the mRNA level we tested 30367 genes, of which 55 were differentially expressed in WKY when comparing expression at 6 weeks to 5 months of age. In SHR, a much larger set of 313 genes were differentially expressed upon maturation, representing approximately 1% of the genes probed on the microarray (Fig 2C). Among the age-related genes, 31 genes were shared between WKY and SHR (Fig 2C). Genes specific for maturation of either WKY or SHR vessels (comparing the expression at 5 months vs. 6 weeks) are listed in Table 3. Comparison of WKY to SHR showed that at 6 weeks of age 87 genes were differentially expressed. At 5 months of age, 115 genes were differentially expressed. Of these, 39 genes were differentially expressed at both time points (Fig 2D). A list of the most strongly up- and downregulated genes that were present at both ages, in SHR as compared to WKY, is shown in Table 3.

**Table 3:** List of most strongly up- and down-regulated mRNAs.

Systematic name	Gene Symbol	Gene name	Fold change			
			**array**		**PCR**	**Ratio**
			**WKY**	**SHR**	**SHR**	**SHR/WKY**
**Specific for WKY maturation**						
NM_031116	*Ccl5*	chemokine (C-C motif) ligand 5	**6.9**	**2.1**		**0.3**
			****	****		****
**Specific for SHR maturation**			****	****		****
NM_017210	*Dio3*	deiodinase, iodothyronine, type III	**1.5**	**7.2**	**2.3**	**4.7**
NM_022604	*Esm1*	endothelial cell-specific molecule 1	**2.4**	**8.8**	**2.1**	**3.7**
NM_021586	*Ltbp2*	latent transforming growth factor beta binding protein 2	**0.8**	**3.0**		**3.6**
NM_031598	*Pla2g2a*	phospholipase A2, group IIA	**2.5**	**8.7**		**3.5**
NM_133306	*Olr1*	oxidized low density lipoprotein (lectin-like) receptor 1	**1.6**	**5.0**		**3.1**
ENSRNOT00000030320	*Mmrn1*	multimerin 1	**0.9**	**0.3**		**0.3**
NM_019157	*Aqp7*	aquaporin 7	**1.2**	**0.4**		**0.3**
NM_012789	*Dpp4*	dipeptidylpeptidase 4	**1.1**	**0.3**		**0.3**
NM_134349	*Mgst1*	microsomal glutathione S-transferase 1	**1.0**	**0.3**		**0.3**
NR_027324	*H19*	H19, imprinted maternally expressed transcript	**0.3**	**0.1**		**0.3**
NM_012777	*Apod*	apolipoprotein D	**0.6**	**0.1**		**0.2**
NM_030865	*Myoc*	myocilin	**0.9**	**0.2**		**0.2**
	**		****	****	****	
** **SHR vs. WKY**			**array**		**PCR**	
	**		**6 weeks**	**5 months**	**5 months**	
NM_017133	*Thbs4*	thrombospondin 4	**4.2**	**9.2**	14.1*	
NM_001008833	*RT1-CE10*	RT1 class I, locus CE10	**5.6**	**6.4**		
NM_001257345	*Pcmtd1*	protein-L-isoaspartate (D-aspartate) O-methyltransferase domain containing 1	**5.5**	**4.7**	**0.3**	
NM_001107564	*Ano1*	anoctamin 1, calcium activated chloride channel	**2.8**	**3.5**	**7.7**	
NM_001039197	*Tor1b*	torsin family 1, member B	**2.0**	**3.0**		
NM_053535	*Enpp1*	ectonucleotide pyrophosphatase/phosphodiesterase 1	**3.2**	**2.9**	**0.7**	
NM_198791	*Tlr3*	toll-like receptor 3	**2.7**	**2.7**		
NM_001008841	*RT1-CE3*	RT1 class I, locus CE3	**3.0**	**2.5**		
NM_001012174	*Fkbp5*	FK506 binding protein 5	**3.4**	**2.3**		
NM_001012218	*Trim55*	tripartite motif-containing 55	**0.3**	**0.3**		
NM_001008858	*RT1-T24-1*	RT1 class I, locus T24, gene 1	**0.3**	**0.3**		
NM_001008843	*RT1-CE5*	RT1 class I, locus CE5	**0.4**	**0.2**		
NM_001025038	*Mettl24*	methyltransferase like 24	**0.3**	**0.1**	5.8 E-6*	
NM_001004084	*RT1-Bb*	RT1 class II, locus Bb	**0.2**	**0.1**		
NM_001013853	*LOC287167*	globin, alpha	**0.1**	**0.1**	4.7 E-6*	
NM_001107284	*Slitrk5*	SLIT and NTRK-like family, member 5	**0.1**	**0.1**	1.8 E-5*	
NM_001127530	*LOC302022*	similar to nidogen 2 protein	**0.2**	**0.1**	**0.5**	

Upper part: genes specifically associated with maturation in either WKY or SHR (adjusted P-value <0.05). Fold changes (5 months vs. 6 weeks) are listed, as well as the ratio SHR/WKY). Lower part: differentially expressed between WKY and SHR at both ages (6 weeks and 5 months). Fold change obtained during PCR confirmation is also shown (* p <0.05, n = 5–6).

Further confirmation of some of the top up- or downregulated miRNA and mRNA from our microarray data was done by RT-PCR in mesenteric arteries from WKY/NTac and SHRSP/A3NCrl (Tables 2 and 3). Most differences could be confirmed (Tables 2 and 3).

### IPA analysis

To elucidate molecular networks and cellular functions enriched for differentially expressed miRNAs and mRNAs, we used the IPA software package. The miRNAs that were differentially expressed with age in WKY involved molecular functions related to cell cycle and cellular development, growth, proliferation, death or movement. In SHR, a much larger number of miRNAs were found to be upregulated upon maturation, involving more genes in the same clusters from WKY (e.g. 24 versus 17 genes in cellular development). In addition, new clusters appeared: inflammatory diseases, connective tissue disorders and metabolic diseases. The comparison between SHR and WKY showed the same molecular and cellular functions as described above, with more miRNAs involved at 5 months than at 6 weeks (S1 Table).

The analysis of the mRNAs differentially expressed in WKY maturation revealed functions related to cellular processes like growth, proliferation and movement. The top networks were related to the same cellular functions but also to connective tissue disorders (S2 Table). In SHR, the differentially expressed age-related genes were partly involved in the same cellular functions as WKY. However, also here, distinct functions and networks were observed that were specific for SHR maturation. For the mRNA analysis, these were related to metabolism and cardiovascular system development and function. In the comparison of SHR and WKY, the differentially expressed genes at 6 weeks were involved in cellular functions related to lipid metabolism, small molecule biochemistry, molecular transport, and cellular movement or proliferation. However, at 5 months, the biochemical and metabolic groups were no longer differentially expressed. Groups that appeared here relate to cell signaling, morphology and cell death. In the top networks as well as in the top physiological and developmental functions, a large number of genes were related to cardiovascular system development and function (S3 Table).

### *Thbs4* and ER stress response genes

**Fig 3:**
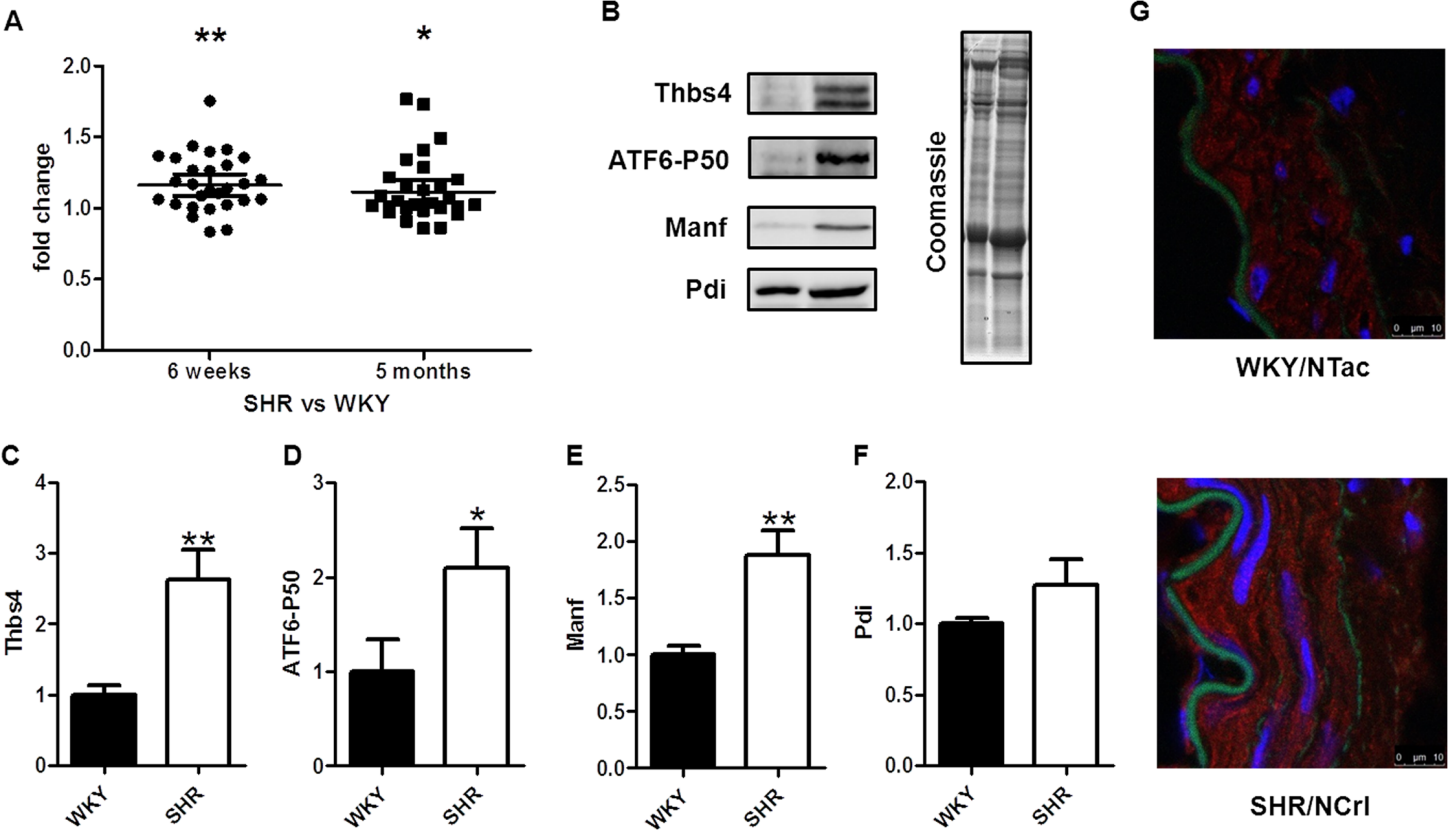
Thrombospondin 4 and ER stress related genes in SHR vessels. Fold changes of genes related to ER stress in the array data in SHR vs WKY at 6 weeks and 5 months (A). Image of a western blot with WKY (left) and SHR (right) aorta samples (B) and quantifications for thrombospondin 4 (THBS4) (C), the activated form of the transcription factor 6α (ATF6α) (D), mesencephalic astrocyte-derived neurotrophic factor (MANF) (E) and protein disulfide isomerase (PDI) (F) (n = 5). Localization of THBS4 by staining in mesenteric artery cross sections: THBS4 (red), nuclei (blue), elastic internal lamina (green) (G). (*p<0.05; **p<0.01; ***p<0.001).

Since thrombospondin 4 (*Thbs4*) was the most overexpressed gene in the comparison between SHR and WKY at both ages, it emerged as an interesting target for further study. Other studies have shown that this protein is involved, among other processes, in the endoplasmic reticulum (ER) stress response, by activating the transcription factor 6α (ATF6α), one of the main mediators in this process [15]. We checked the mRNA levels of both *Atf6α* and a large number of downstream targets [12] in the microarray data and found a significant differential expression in SHR vs WKY at both 6 weeks (P = 0.003) and 5 months (P = 0.04) (Fig 3A). Since changes at the mRNA level do not necessarily translate to differences at the protein level, we analyzed aortic homogenates that were obtained from the same animals by western blotting. These data confirmed our microarray results, as we found a significant increase at the protein level of THBS4, the cleaved (active) form of ATF6α, and one of its downstream targets, mesencephalic astrocyte-derived neurotrophic factor (MANF) in SHR as compared to WKY (Fig 3C–3E). For another downstream target, the protein disulfide isomerase (PDI), there was a tendency of upregulation although this was not significant (Fig 3F). In sections of mesenteric arteries, THBS4 was localized in the cytoplasm of smooth muscle cells (Fig 3G).

## Discussion

The objective of the current study was to investigate gene expression in the development of hypertension in mesenteric arteries of SHR as compared to WKY by miRNA and mRNA microarray analysis and bioinformatical tools. Three sublines of SHR and WKY were used to identify genes with consistent up or down regulation in hypertension, and two time points (6 weeks and 5 months) were analyzed. Limitations of our study were that we used mesenteric arteries (approximately 350 micrometer in diameter) that are at the upper end of the range of vessels that contribute to peripheral resistance [16]. In addition, it should be noted that differences in gene expression are likely to exist between vascular beds and perhaps even among vessels from different branching orders, as large and small resistance arteries are known to be heterogeneous [17].

### Maturation in WKY and SHR

Principal component analysis (PCA) showed that gene expression profiles are strongly age-related. The maturation of WKY and SHR vessels showed considerable overlap in terms of miRNA and mRNA expression profiles. Thus, maturation in both WKY and SHR vessels was associated with a prominent increase in expression of the miR-29 family. MiR-29 has previously been related to aging and dilation of the aorta, associated with down-regulation of extracellular matrix molecules [18]. This is confirmed by a recent study where miR-29b was related to a reduction in extracellular matrix proteins as shown by a proteomic approach [19]. In the present study the mRNA microarray experiment showed that many of the targets of miR-29 were indeed significantly down-regulated, both in WKY and SHR. In fact, expression of many extracellular matrix proteins such as collagens and elastin decreased with vascular maturation. Most specific for maturation of SHR vessels at the miRNA level was the up-regulation of miR-132-3p. MiR-132 is one of the microRNAs that is up-regulated by angiotensin II. Its upregulation is related to cell cycle, motility, and cardiovascular functions [20], which is in agreement with the IPA analysis where the same pathways were identified. This miRNA therefore seems an interesting target for further studies.

Analysis of differentially expressed genes with IPA software showed that among the age related genes for WKY and SHR, the number of genes involved in functions like proliferation, cell movement or cardiovascular system development and function that reached statistical significance, was larger in SHR. Specifically for SHR maturation a group of genes related to metabolism of lipids, drugs and carbohydrates as well as small molecule biochemistry emerged from the analysis. This includes genes such as phospholipase A2 group IIA, oxidized low density lipoprotein receptor 1 and apolipoprotein D, suggesting alterations in metabolism in SHR as compared to WKY. In addition to these genes, interesting new candidates specific for maturation of SHR emerged from the mRNA analysis. The most strongly upregulated was the type III deiodinase (*Dio3*). This is a fetally expressed thyroid hormone-inactivating enzyme associated with cell proliferation, which was recently shown to be up-regulated in the spared part of the myocardium after infarction [21]. Its main substrate, the triiodothyronine (T3 thyroid hormone), has been reported to have effects in the relaxation of mesenteric arteries [22]. The second specific gene was *Esm1* or endocan, which recently has been proposed as new marker in essential hypertension [23]. Thus, a number of interesting miRNAs, genes and processes such as proliferation, migration, and metabolism are linked to maturation, with some being specific for SHR.

Both at the miRNA and mRNA level a more extensive age-related change in expression profile was noted in SHR as compared to WKY. Based on this, one could argue that hypertension is associated with the up- or downregulation of many more genes during development. However, another possible reason for this difference could be a larger heterogeneity among WKY sublines. This can be seen in the PCA and clustering for both miRNAs and mRNAs, where SHR sublines appear more homogeneous. Thus, at the miRNA level the WKY/NHsd separated from the other WKY sublines. At the mRNA level, the WKY/NTac at 6 weeks of age clustered with the 5 months old WKY groups. In a previous study we observed a similar high variability of vascular function and structure in these sublines [8]. The heterogeneity in WKY has also been pointed out by other groups, at the genetic [24] and phenotypic [25] level. This seems to be caused by the distribution of the rats before they were fully inbred, contrary to SHR animals [24].

### Comparison of WKY to SHR

At the miRNA level, a direct comparison between SHR and WKY yielded remarkably few differences. Again, this may relate to heterogeneity among sublines of rats. Analysis of the comparison between SHR and WKY revealed a number of molecules involved in cell growth and proliferation pathways, suggesting that these differentially expressed miRNAs could be involved in the regulation of proliferation in hypertension. This is consistent with the up-regulation of miR-31a-3p and -5p and down-regulation of miR-146a-5p in SHR as compared to WKY. Recent work shows that miR-31 controls smooth muscle phenotype and proliferation [26]. MiR-31 is an abundant miRNA in vascular smooth muscle cells, and its expression is significantly increased in proliferative smooth muscle cells and in vascular walls with neointimal growth [26]. Thus, the increased expression of miR-31a-3p/5p in the present study seems to be in accordance with the increased wall thickness and number of smooth muscle layers in SHR in our previous work [8]. It should however be noted that the increase in expression of miR-31a-3p/5p may, in part, be a consequence of differences in the ratio of smooth muscle cells over endothelial cells and/or other cell types. MiR-146a, which was downregulated in the current study, has also been related to cardiovascular disease. However, in contrast to our study, miR-146a was found to be up-regulated in human atherosclerosis [27]. In vitro data suggest that this microRNA increases smooth muscle proliferation [28]. In endothelial cells, down-regulation of miR-146a is associated with production of extracellular matrix proteins in the context of diabetes [29].

Regarding mRNA expression, in the comparison between SHR and WKY, the top gene network was related to metabolism. Also in this comparison, a large number of genes was involved in cellular growth and proliferation or cellular movement, as seen in the SHR maturation. This is in agreement with the increase in wall thickness that we previously found in SHR [8]. It is also remarkable that a number of genes that are differentially expressed were related to the immune system. Although at first sight this could suggest an ongoing inflammatory processes, typical markers of inflammation and leukocyte infiltration such as CD68 did not emerge in our study. It is therefore more likely that differences in the expression of these immune related genes are consequence of the genetic differences between WKY and SHR.

### ER stress

The most significantly upregulated gene in SHR vessels as compared to WKY at both ages was thrombospondin 4 (*Thbs4*). This protein is frequently considered to be an extracellular matrix protein. It was recently identified as a mechano-signaling molecule in the heart [30]. It has been shown to be up-regulated in the heart during pressure overload, where it is expressed in various tissues including blood vessels [31]. In the latter study, it was shown to limit inflammation and fibrosis in the heart, indicating that it plays an important role in matrix remodeling. However, in our hands, immunostaining revealed that THBS4 is mainly located intracellularly. This is more consistent with a second important role attributed to THBS4, the ER stress response via ATF6α in heart [15]. We therefore focused on this pathway, and further investigated ATF6α and its downstream targets.

ER stress leads to misfolding and aggregation of proteins in the ER lumen. Recently, several studies have pointed out a link between hypertension and ER stress. Thus, ER stress inhibitor treatment decreased blood pressure, as well as aortic apoptosis, collagen content and fibrosis in aortas of Ang II infused Sprague Dawley rats [32]. The ER stress inducer tunicamycin increases blood pressure and vascular smooth muscle contractility [33]. In SHR, suppression of ER stress normalizes endothelium-derived contracting factors (EDCF)-mediated contractions in the aorta of the SHR [34]. The strong up-regulation of *Thbs4* led us to analyze 23 targets of ATF6α which indeed showed a clear pattern of up-regulation of the whole ER stress signaling pathway. Further analysis of the expression of THBS4, the cleaved form of ATF6α, and two of its targets by western blot confirmed the up-regulation of this pathway at the protein level in hypertension. In addition to this, another of the top upregulated genes in the array, the *Tor1b* (torsin family 1, member B) belongs to a family of proteins present in the membrane of the ER that has also been related to ER stress [35–38]. Thus, our data support the hypothesis that ER stress plays an important role in hypertension, particularly at the level of the resistance arteries.

### Conclusions

Taken together, miRNA and mRNA expression analysis identified a large number of miRNAs and genes in resistance arteries that are differentially expressed upon maturation, some of which are specifically related to the development of hypertension. Those genes are mainly involved in extracellular matrix, vascular tone, metabolism, and in cell proliferation and growth processes. We report new miRNAs and genes of interest for further research in hypertension. One of these is *Thbs4*, which appears to play a role in ER stress associated with hypertension

## Supporting Information

S1 Table IPA results for miRNA microarray data. Groups with the most relevant number of miRNAs for the different categories in the IPA core analysis. # molecules is the number of miRNAs involved in a certain group. In the top networks, the score is calculated by the software using Fisher’s exact test and defined as: p-score = -log10(p-value).
(PDF)

S2 Table IPA results for mRNA microarray data in 5 months vs 6 weeks comparison. Groups with the most relevant number of mRNAs for the different categories in the IPA core analysis for 5 months vs 6 weeks comparison in WKY and SHR animals. # molecules is the number of mRNAs involved in a certain group. In the top networks, the score is calculated by the software using Fisher’s exact test and defined as: p-score = -log10(p-value).
(PDF)

S3 Table IPA results for mRNA microarray data in SHR vs WKY comparison. Groups with the most relevant number of mRNAs for the different categories in the IPA core analysis for SHR vs WKY comparison at both ages. # molecules is the number of miRNAs involved in a certain group. In the top networks, the score is calculated by the software using Fisher’s exact test and defined as: p-score = -log10(p-value).
(PDF)
