# Supplementary material for: Gene Expression and MicroRNA Expression Analysis in Small Arteries of Spontaneously Hypertensive Rats. Evidence for ER Stress
Source: PLoS One. 2015 Sep 10;10(9):e0137027. doi: 10.1371/journal.pone.0137027 (PMC4565692; doi:10.1371/journal.pone.0137027)
Supplement: S1 Table — Groups with the most relevant number of miRNAs for the different categories in the IPA core analysis. # molecules is the number of miRNAs involved in a certain group. In the top networks, the score is calculated by the software using Fisher’s exact test and defined as: p-score = -log10(p-value). (PDF) [file pone.0137027.s001.pdf]

Diseases and Disorders

SHR vs WKY 6 weeks		
Name	p-value	# Molecules
Cardiovascular Disease	3.78E-07 - 4.89E-02	5
Connective Tissue Disorders	3.78E-07 - 1.23E-02	9
Dermatological Diseases and Conditions	3.78E-07 - 2.09E-03	8
Inflammatory Disease	3.78E-07 - 1.23E-02	7
Skeletal and Muscular Disorders	3.78E-07 - 1.23E-02	5

SHR vs WKY 5 months		
Name	p-value	# Molecules
Cancer	1.27E-18 - 4.64E-02	22
Organismal Injury and Abnormalities	1.27E-18 - 4.64E-02	20
Reproductive System Disease	1.27E-18 - 3.99E-02	18
Connective Tissue Disorders	1.99E-17 - 1.90E-02	13
Endocrine System Disorders	4.82E-15 - 6.76E-03	18

Molecular and Cellular Functions

SHR vs WKY 6 weeks		
Name	p-value	# Molecules
Cell Death and Survival	1.04E-03 - 1.96E-02	3
Cell-To-Cell Signaling and Interaction	1.04E-03 - 1.04E-03	3
Cellular Assembly and Organization	1.04E-03 - 1.04E-03	1
Cellular Development	1.04E-03 - 3.99E-02	5
Cellular Function and Maintenance	1.04E-03 - 4.79E-02 2	2

SHR vs WKY 5 months		
Name	p-value	# Molecules
Cell Cycle	1.77E-06 - 4.38E-02	7
Cellular Development	5.30E-06 - 3.86E-02	16
Cell-To-Cell Signaling and Interaction	7.91E-05 - 3.08E-02	6
Cellular Growth and Proliferation	7.91E-05 - 3.86E-02	14
Cellular Movement	1.70E-04 - 4.58E-02	7

Physiological system development and function

SHR vs WKY 6 weeks		
Name	p-value	# Molecules
Embryonic Development	1.04E-03 - 1.18E-03	3
Hair and Skin Development and Function	1.04E-03 - 2.07E-02	1
Hematological System Development and Function	1.04E-03 - 4.79E-02	3
Immune Cell Trafficking	1.04E-03 - 1.04E-03	1
Nervous System Development and Function	1.04E-03 - 1.04E-03	1

SHR vs WKY 5 months		
Name	p-value	# Molecules
Hematological System Development and Function	5.30E-06 - 3.47E-02	9
Hematopoiesis	5.30E-06 - 3.47E-02	8
Lymphoid Tissue Structure and Development	5.30E-06 - 2.14E-02	4
Tissue Development	1.06E-05 - 2.94E-02	11
Tissue Morphology	2.98E-04 - 3.99E-02	5

Top Networks

SHR vs WKY 6 weeks	
Associated Network Functions	score
Cancer. Endocrine System Disorders. Gastrointestinal Disease	30
Cell Death and Survival. Cell-To-Cell Signaling and Interaction. Cellular Growth and Proliferation	11
Developmental Disorder. Hereditary Disorder. Skeletal and Muscular Disorders	3
Cardiac Fibrosis. Cardiovascular Disease. Organismal Injury and Abnormalities	3

SHR vs WKY 5 months	
Associated Network Functions	score
Cancer. Organismal Injury and Abnormalities. Reproductive System Disease	42
Cancer. Hematological Disease. Hereditary Disorder	21
Endocrine System Disorders. Gastrointestinal Disease. Metabolic Disease	3
