# Supplementary material for: Gene Expression and MicroRNA Expression Analysis in Small Arteries of Spontaneously Hypertensive Rats. Evidence for ER Stress
Source: PLoS One. 2015 Sep 10;10(9):e0137027. doi: 10.1371/journal.pone.0137027 (PMC4565692; doi:10.1371/journal.pone.0137027)
Supplement: S2 Table — Groups with the most relevant number of mRNAs for the different categories in the IPA core analysis for 5 months vs 6 weeks comparison in WKY and SHR animals. # molecules is the number of mRNAs involved in a certain group. In the top networks, the score is calculated by the software using Fisher’s exact test and defined as: p-score = -log10(p-value). (PDF) [file pone.0137027.s002.pdf]

Top canonical pathways

WKY 5 months vs 6 weeks		
**Name**	**p-value**	**ratio**
Hepatic Fibrosis / Hepatic Stellate Cell Activation	1.63E-13	36/197 (0.183)
Atherosclerosis Signaling	7.81E-11	25/123 (0.203)
Intrinsic Prothrombin Activation Pathway	1.53E-08	11/29 (0.379)
Granulocyte Adhesion and Diapedesis	1.77E-07	25/177 (0.141)
Mitotic Roles of Polo-Like Kinase	6.65E-07	14/66 (0.212)

SHR 5 months vs 6 weeks		
**Name**	**p-value**	**ratio**
Hepatic Fibrosis / Hepatic Stellate Cell Activation	3.42E-13	41/197 (0.208)
Atherosclerosis Signaling	4.09E-11	29/123 (0.236)
Granulocyte Adhesion and Diapedesis	2.58E-07	29/177 (0.164)
Glutathione-mediated Detoxification	1.77E-06	10/28 (0.357)
Triacylglycerol Biosynthesis	2.29E-06	11/35 (0.314)

Top Networks

WKY 5 months vs 6 weeks	
**Associated Network Functions**	**score**
Cell Cycle, Cellular Assembly and Organization, DNA Replication, Recombination, and Repair	37
Cell Cycle, Cellular Assembly and Organization, DNA Replication, Recombination, and Repair	35
Tissue Development, Cancer, Cell-To-Cell Signaling and Interaction	33
Connective Tissue Disorders, Inflammatory Disease, Neurological Disease	33
Connective Tissue Disorders, Dermatological Diseases and Conditions, Organismal Injury and Abnormalities	31

SHR 5 months vs 6 weeks	
**Associated Network Functions**	**score**
Carbohydrate Metabolism, Drug Metabolism, Molecular Transport	41
Cell Cycle, Cellular Assembly and Organization, DNA Replication, Recombination, and Repair	34
Molecular Transport, Visual System Development and Function, Cardiac Arrythmia	32
Cell-To-Cell Signaling and Interaction, Cell Signaling, Cardiovascular System Development and Function	32
Organ Morphology, Organismal Development, Reproductive System Development and Function	30

Molecular and Cellular Functions

WKY 5 months vs 6 weeks		
**Name**	**p-value**	**# molecules**
Cellular Growth and Proliferation	3.61E-22 - 2.72E-05	347
Cellular Movement	1.70E-18 - 2.68E-05	240
Cell Cycle	7.58E-17 - 2.27E-05	152
Cell Morphology	6.43E-16 - 2.47E-05	228
Cell Death and Survival	1.48E-15 - 1.55E-05	302

SHR 5 months vs 6 weeks		
**Name**	**p-value**	**# molecules**
Cellular Movement	5.04E-35 - 2.09E-07	321
Cellular Growth and Proliferation	7.05E-30 - 1.51E-07	459
Cell Morphology	9.52E-29 - 1.88E-07	348
Lipid Metabolism	1.23E-23 - 1.95E-07	231
Small Molecule Biochemistry	1.23E-23 - 2.02E-07	300

Physiological system development and function

WKY 5 months vs 6 weeks		
**Name**	**p-value**	**# Molecules**
Organismal Survival	1.21E-16 - 1.55E-04	144
Cardiovascular System Development and Function	1.59E-15 - 2.51E-05	159
Organismal Development	1.59E-15 - 2.51E-05	244
Tissue Development	1.32E-14 - 2.72E-05	291
Tissue Morphology	2.01E-14 - 2.14E-05	227

SHR 5 months vs 6 weeks		
**Name**	**p-value**	**# Molecules**
Cardiovascular System Development and Function	1.34E-34 - 2.08E-07	285
Organismal Development	1.34E-34 - 1.51E-07	417
Tissue Morphology	7.91E-26 - 1.88E-07	344
Organismal Survival	4.09E-24 - 6.67E-08	323
Immune Cell Trafficking	1.08E-22 - 2.10E-07	191
