# Supplementary material for: Gene Expression and MicroRNA Expression Analysis in Small Arteries of Spontaneously Hypertensive Rats. Evidence for ER Stress
Source: PLoS One. 2015 Sep 10;10(9):e0137027. doi: 10.1371/journal.pone.0137027 (PMC4565692; doi:10.1371/journal.pone.0137027)
Supplement: S3 Table — Groups with the most relevant number of mRNAs for the different categories in the IPA core analysis for SHR vs WKY comparison at both ages. # molecules is the number of miRNAs involved in a certain group. In the top networks, the score is calculated by the software using Fisher’s exact test and defined as: p-score = -log10(p-value). (PDF) [file pone.0137027.s003.pdf]

Top canonical pathways

SHR vs WKY 6 weeks		
Name	p-value	ratio
LXR/RXR Activation	1.54E-06	18/121 (0.149)
OX40 Signaling Pathway	2.32E-06	15/89 (0.169)
Complement System	4.12E-06	9/33 (0.273)
Allograft Rejection Signaling	3.62E-05	13/86 (0.151)
Serine Biosynthesis	6.03E-04	3/5 (0.6)

SHR vs WKY 5 months		
Name	# molecules	p-value
Atherosclerosis Signaling	6.01E-10	28/123 (0.228)
Granulocyte Adhesion and Diapedesis	4.37E-09	33/177 (0.186)
Agranulocyte Adhesion and Diapedesis	6.64E-09	34/189 (0.18)
Hepatic Fibrosis / Hepatic Stellate Cell Activation	6.64E-08	33/197 (0.168)
Altered T Cell and B Cell Signaling in Rheumatoid Arthritis	5.70E-05	16/88 (0.182)

Molecular and Cellular Functions

SHR vs WKY 6 weeks		
Name	p-value	# Molecules
Cellular Movement	5.96E-20 - 1.55E-04	212
Lipid Metabolism	1.11E-16 - 1.42E-04	167
Small Molecule Biochemistry	1.11E-16 - 1.94E-04	237
Cellular Growth and Proliferation	1.45E-15 - 2.15E-04	292
Molecular Transport	3.55E-15 - 1.79E-04	222

SHR vs WKY 5 months		
Name	p-value	# Molecules
Cellular Movement	2.30E-25 - 4.56E-06	307
Cellular Growth and Proliferation	4.93E-24 - 5.04E-06	442
Cell Death and Survival	6.99E-19 - 4.09E-06	415
Cell-To-Cell Signaling and Interaction	2.09E-18 - 3.99E-06	220
Cell Morphology	1.14E-17 - 1.80E-06	316

Top Networks

SHR vs WKY 6 weeks	
Associated Network Functions	score
Cell-To-Cell Signaling and Interaction, Cell Signaling, Cardiovascular System Development and Function	39
Developmental Disorder, Hereditary Disorder, Immunological Disease	34
Cellular Development, Connective Tissue Development and Function, Digestive System Development and Function	30
Organismal Development, Carbohydrate Metabolism, Molecular Transport	29
Cell Death and Survival, Connective Tissue Disorders, Developmental Disorder	29

SHR vs WKY 5 months	
Associated Network Functions	score
Lipid Metabolism, Small Molecule Biochemistry, Vitamin and Mineral Metabolism	38
Developmental Disorder, Hereditary Disorder, Immunological Disease	38
Cell Cycle, Cellular Assembly and Organization, DNA Replication, Recombination, and Repair	33
Hematological System Development and Function, Cell Death and Survival, Connective Tissue Disorders	31
Cardiovascular System Development and Function, Organismal Development, Cardiovascular Disease	31

Physiological system development and function

SHR vs WKY 6 weeks		
Name	p-value	# Molecules
Immune Cell Trafficking	1.21E-16 - 1.55E-04	144
Hematological System Development and Function	4.13E-14 - 1.55E-04	207
Tissue Morphology	3.24E-13 - 1.51E-04	211
Cardiovascular System Development and Function	2.56E-11 - 1.75E-04	157
Endocrine System Development and Function	8.10E-11 - 2.16E-08	41

SHR vs WKY 5 months		
Name	p-value	# Molecules
Tissue Morphology	6.11E-24 - 3.19E-06	295
Cardiovascular System Development and Function	8.70E-19 - 5.04E-06	236
Hematological System Development and Function	1.17E-18 - 4.56E-06	311
Organismal Development	1.75E-17 - 5.04E-06	330
Immune Cell Trafficking	5.44E-16 - 4.56E-06	182
